# Chronic Maternal Depression Is Associated with Reduced Weight Gain in Latino Infants from Birth to 2 Years of Age

**DOI:** 10.1371/journal.pone.0016737

**Published:** 2011-02-23

**Authors:** Janet M. Wojcicki, Katherine Holbrook, Robert H. Lustig, Elissa Epel, Aaron B. Caughey, Ricardo F. Muñoz, Stephen C. Shiboski, Melvin B. Heyman

**Affiliations:** 1 Division of Pediatric Gastroenterology and Nutrition, Department of Pediatrics, University of California San Francisco, San Francisco, California, United States of America; 2 Division of Pediatric Endocrinology, Department of Pediatrics, University of California San Francisco, San Francisco, California, United States of America; 3 Department of Obstetrics and Gynecology, University of California San Francisco, San Francisco, California, United States of America; 4 Department of Psychiatry, University of California San Francisco, San Francisco, California, United States of America; 5 Department of Epidemiology and Biostatistics, University of California San Francisco, San Francisco, California, United States of America; Indiana University, United States of America

## Abstract

**Background:**

Latino children are at increased risk for mirconutrient deficiencies and problems of overweight and obesity. Exposures in pregnancy and early postpartum may impact future growth trajectories.

**Objectives:**

To evaluate the relationship between prenatal and postnatal maternal depressive symptoms experienced in pregnancy and infant growth from birth to 2 years of age in a cohort of Latino infants.

**Methods:**

We recruited pregnant Latina mothers at two San Francisco hospitals and followed their healthy infants to 24 months of age. At 6, 12 and 24 months of age, infants were weighed and measured. Maternal depressive symptoms were assessed prenatally and at 4-6 weeks postpartum. Women who had high depressive symptoms at both time periods were defined as having chronic depression. Logistic mixed models were applied to compare growth curves and risk for overweight and underweight based on exposure to maternal depression.

**Results:**

We followed 181 infants to 24 months. At 12 and 24 months, respectively, 27.4% and 40.5% were overweight, and 5.6% and 2.2% were underweight. Exposure to chronic maternal depression was associated with underweight (OR = 12.12, 95%CI 1.86-78.78) and with reduced weight gain in the first 2 years of life (Coef = -0.48, 95% CI -0.94—0.01) compared with unexposed infants or infants exposed to episodic depression (depression at one time point). Exposure to chronic depression was also associated with reduced risk for overweight in the first 2 years of life (OR 0.28, 95%CI 0.03-0.92).

**Conclusions:**

Exposure to chronic maternal depression in the pre- and postnatal period was associated with reduced weight gain in the first two years of life and greater risk for failure to thrive, in comparison with unexposed infants or those exposed episodically. The infants of mothers with chronic depression may need additional nutritional monitoring and intervention.

## Introduction

Maternal prenatal depression can have significant and critical negative impact in fetal development and occurs in approximately 7 to 13% of childbearing women [1], [2]. Depression during pregnancy can lead to greater risk for preterm delivery and lower birthweight [3], [4]. Additionally, during the postnatal period, infant exposure to postpartum depression is associated with increased risk for attenuated growth during the first 2 years of life [5], [6]. The hypothalamic-pituitary-adrenal (HPA) axis can become dysregulated in infancy by early exposure to stressors including maternal depression, which may sensitize children's responses to subsequent stress exposure and could potentially explain disordered eating, included stress-related eating and inability to self-regulate [7].

In addition to the above described associations between prenatal and postnatal depression, lower birthweight, and failure to thrive, maternal postnatal depressive symptoms have also been associated with risk for pediatric overweight in the first 2 years of life. In a cross-sectional study from Brazil, Surkan et al, reported that postnatal depressive symptoms (from 6-24 months) are associated with increased risk for overweight and obesity for children between 6 and 24 months of age [8].

Studies suggest that the time period of exposure is likely important in determining childhood outcome. A recent US-based study found that children exposed to antenatal maternal depression have a lower BMI z-score at 3 years of age, while those exposed to postnatal maternal depression at 6 months have higher overall adiposity [9]. Meanwhile, Grote et al, in a European longitudinal study, did not find growth differences at 2 years of age in children exposed to postnatal depression at 2, 3 and 6 months [10]. Similarly, Ajslev et al. 2010 did not find any association between exposure to postpartum depression at 6 months of age and overweight at age 7 [11]. The severity and duration of the exposure may also determine child outcome. A study from Pakistan found that infants exposed to both prenatal and postnatal depression had poorer growth until age one with those exposed to chronic maternal depression at greater risk than those exposed to episodic depression [12].

No study to date has evaluated the combined impact of exposure to prenatal and postnatal depression in a population at high risk for future obesity such as Latino infants, in comparison with unexposed infants or those exposed to maternal depressive symptoms either prenatally or postnatally. Such an approach is needed as the infants of women who have more intractable depressive symptoms that span the perinatal period may have different growth trajectories in comparison with infants who do not have these environmental exposures or a more limited form of exposure (either prenatal or postnatal).

The early growth trajectories of Latino children are an important group to assess given the high prevalence of nutritional deficiencies in this population as well as the high risk of future obesity. Latino children have high prevalences of zinc deficiency [13] and low vitamin E intakes [14]. Moreover, Mexican-American children (2–19 years), the largest population group of Latino children in the United States, have a higher prevalence of overweight and obesity (37.0%) compared with non-Hispanic Caucasian/white (33.5%) and African-American children (35.1%) [15]. Mexican-American children are also at a higher risk for severe obesity (>99^th^ percentile), at 5.7%, compared with 3.1% of non-Hispanic whites [16]. Recently, Taveras et al. have argued that population groups with a greater risk of childhood obesity, such as Latinos, may have increased exposure to maternal postnatal depression compared with white children [17]. Given the high prevalence of obesity and other nutritional deficiencies in Latino children, and the importance of evaluating early life risk factors, including the possible impact of developmental programming on a child's future obesity risk, it is important to better understand the impact that exposure to depressive symptoms may have on infant growth.

## Methods

### Participants

Latina women were recruited during the 2^nd^ and 3^rd^ trimesters of pregnancy at prenatal clinics at the University of California, San Francisco (UCSF) Medical Center and San Francisco General Hospital (SFGH) from May 2006 to May 2007, and were followed through labor and delivery until the infants reached 2 year of age. Exclusion criteria included drugs or alcohol abuse, pre-existing diabetes mellitus or gestational diabetes mellitus treated with insulin, polycystic ovarian syndrome, any eating disorders such as bulimia or anorexia nervosa, or any health problems that would influence breast-feeding. Infants at delivery were excluded if they had special care needs, chronic disease, or Apgar scores of less than 7 at 5 minutes. Close to 100% of Latina women were approached in both hospitals as we had a continuous on-site research presence. Approximately 90% of those who met the inclusion criteria for the study agreed to participate.

### Procedures

All procedures were approved by the Committee on Human Research at UCSF and the Institutional Review Board at SFGH. Following informed consent, baseline data and socio-demographics of the participants including age, education, occupation, income, marital status, language use and length of time in the United States were collected. Medical history was also ascertained through chart review and by questionnaire to determine mental health history, including previous diagnosis and/or treatment for depression or anxiety. Maternal pre-pregnancy weight was collected at baseline by self-report. Questions on legal status or migration history were not asked other than length of time in the United States because of the sensitive nature of these questions and the possibility that questioning in this area could compromise the validity of our study or jeopardize the follow-up rate. Additional specifics about our study population are described in Wojcicki et al. [18].

Upon enrollment, mothers were administered the Edinburgh Postpartum Depression Scale (EPDS) [19] and the Center for Epidemiologic Studies Depression Scale (CES-D) [20] to assess for current depressive symptoms. The Mini International Neuropsychiatric Interview (M.I.N.I, version 5.0) [21] was used to evaluate for current major depressive episodes. All interviews were conducted either in either English or Spanish and all measures to assess mental health used had previously been validated in Spanish speaking populations as previously described [18]. If mothers were found to have depressive symptom scores they were referred in pregnancy to a psychiatric obstetrical clinic for further evaluation. In the postpartum period, mothers that had high depressive symptom scores were provided information on where to receive follow-up evaluations and treatment.

At birth, anthropometric measurements of the infant, including infant weight (using standard digital infant scales) and length (using standard tape measurements) were obtained. Gestational age and Apgar scores were recorded. At 4–6 weeks postpartum, participants were contacted by phone and interviewed again for depressive symptoms and clinical depression using the same instruments employed at baseline. Additionally, infant feeding was assessed using a 24 hour dietary recall. At 6, 12 and 24 months post-partum, the infants were weighed and measured. Maternal weight (using a Seca digital scale recording weight to nearest 0.1kg) and height (using a portable stadiometer to nearest 0.1cm) were measured between 12 and 24 months postpartum to calculate body mass index (BMI) (kg/m^2^). Waist circumference in children was measured at 24 months of age in a sub-sample of the cohort (n = 156). A small percentage of the cohort (between 3–5% depending on timepoint) were not available to be measured so weight and height were extracted from the medical record. BMI <25 kg/m^2^ was categorized as normal/underweight, 25≤ BMI <30 was categorized as overweight, and BMI ≥30 was categorized as obese, using standard definitions from the World Health Organization [22].

### Statistical Analysis

#### Predictor and outcome variables

The main predictor of interest was depressive symptoms experienced either prenatally and/or at 4–6 weeks postpartum described more in detail below. The main outcome was child weight-for-length z-score at 6,12, and 24 months of age. Actual ages at post-partum visits varied somewhat from these values, so analyses used ages from observed visits. Analyses based on the grouped ages (0, 6, 12 and 24 months) yielded very similar results (not shown). Secondary outcomes of interest included risk for underweight/failure to thrive (<5^th^ percentile weight-for-age), overweight (≥85^th^ percentile weight-for-length or body mass index) and obese (≥95^th^ percentile weight/height or body mass index) as described in more detail below. Additional predictor variables considered for multivariate regression models included infant birth weight z-score or birth weight-for-length z-score, any breast-feeding at 6 months of age, maternal postnatal (12–24 months) BMI, maternal ethnicity (Central American versus Mexican), maternal age and gestational age. Additional details about specific analyses are described below. Birthweight andg birthweight for length Z scores were adjusted for in the analyses because our primary outcome of interest was weight gain from 6 months to 2 years of age in relation to prenatal and postnatal maternal depression exposures.

#### Depressive Symptoms

Exposure to pre- or postnatal depressive symptoms were defined by: 1) CESD ≥16; 2) EPDS ≥13; or 3) having a major depressive episode or dysthymia as per the MINI. A high depression symptom score was defined as a high score on one of the above measures prenatally or at the 4–6 week timepoint. Chronic depression was defined as having a high score at both time points (including a possible diagnosis of clinical depression as per the MINI at either time point) and episodic depression was defined as having depression at one time point (including a possible diagnosis of clinical depression as per the MINI at either time point). We also evaluated the relationship between weight trajectories and not ever having depressive symptoms compared with having depressive symptoms only in the prenatal period, only in the postnatal period, and at both time-points.

#### Weight Variables

Overweight (≥85^th^ percentile BMI or weight/length) and obesity (≥95^th^ percentile BMI or weight/length) up until 24 months were defined by the National Center for Health Statistics, Center for Disease Control and Prevention weight-for-length and body mass index growth curves [23]. Underweight/failure to thrive <5^th^ percentile weight) was defined using the same growth curves. Z-scores were similarly defined from the National Center for Health Statistics, Center for Disease Control and Prevention [23]. High waist circumference scores (>90^th^ percentile) was defined using the cut-points described by Fernandez et al. [24].

#### Statistical Analyses

Chi-squared tests of association, analysis of variance (ANOVA) and Student's t-tests were applied to evaluate the relationships between exposure to prenatal and/or postnatal depression and weight/length percentiles, risk for underweight and risk for overweight and obesity. Mixed effects linear regression models were used to evaluate differences in weight-for-length z-scores over the two-year period in relation exposure to maternal depression adjusting for the potential confounders described above. Models included random intercepts, and random slopes for time effects. Differences in individual weight trajectories between groups reporting different levels of depression were evaluated via inclusion of appropriate interaction terms in fitted models. Mixed effects logistic regression models were used to determine risk factors for failure to thrive, overweight and obesity. Time effects were modeled using the same approach described for linear models. Outcomes for logistic and linear models only included data from visits occurring post-birth as our major predictors included postnatal depression at 4-6 weeks postpartum. As birthweight occurred prior to postnatal depression, it was not possible to include birthweight in the longitudinal analysis as an outcome because of the temporal sequence of events.

Only women who had all data on the selected variables were included in the multivariate models. Data were entered into Excel, and subsequent analyses were conducted using Stata 11.0 (Stata Corporation, College Station, TX, USA).

#### Missing Data

In order to assess the possible role of missing data on our estimates, multiple imputation methods were used in Stata 9.0 using the command *ice*. Five imputed datasets were created and the command mim was used to run mixed logistic and mixed regression models with complete datasets similar to the models described above. Approximately 8% of the data was missing from the bivariate models and 15% from the multivariate models. The bivariate and multivariate results were similar for our primary outcomes comparing the unimputed and imputed models.

## Results

We enrolled 201 women in the cohort^18^ and followed 175 women to 6 months of age and 174 to 12 months of age and 181 to 2 years of age. The majority of our cohort (61.2% [123/201]) was Mexican ethnicity; the remainder was primarily of Central American origin (El Salvadorian, Guatemalan and Honduran). Most women (92.0%) were participants in the Women, Infants and Children's Program (WIC), 31.0% were married, and 52.0% were living with partner. Our participants tended to have a high school diploma or less (76.0%), and 92.9% cited Spanish as their primarily language. The mean number of other children in the household was 0.83±0.99. The majority of the infants in the cohort were born vaginally (85.6%). Mean gestational age was 39.3 weeks, and mean birthweight was 3.37±0.48 kilograms as described in Wojcicki et al. [18].

### Prenatal and Postnatal Depression

Prenatally, 28.9% of participants had depressive symptoms or clinical depression. At 4–6 weeks post-partum this number declined to 15.7%. Depressive symptoms were evident in 22.5% of women at the prenatal or 4–6 week time point, and 11.0% had chronic depression at the prenatal and 4–6 week timepoint. A smaller 4.0% (8/201) had clinical depression prenatally and 4.3% at 4–6 weeks but only one of these eight had clinical depression at both time-points.

### Infant Weight Parameters at 6, 12 and 24 months and Maternal Postnatal BMI

At birth, 7.4% of infants were overweight. By 6 months of age this increased to 26.1%, at 12 months to 27.6%, and at 2 years to 40.5%. At birth, 2.1% of infants were obese; this percentage increased to 11.1% at 6 months, 12.6% at 12 months, and 20.2% at 2 years of age. At birth, an expected 5.2% were <5^th^ percentile for weight. By 2 years of age, only 2.2% were <5^th^ percentile for weight (Table 1). At 24 months, 8.3% of those measured had a waist circumference >90^th^ percentile [24]. Mean maternal postnatal BMI was 28.3±6.3 and 67.5% were overweight and 33.5% were obese.

**Table 1:** Weight Parameters and Breastfeeding Frequencies at Study Timepoints.

Mean ±SD or % (N/Total)	
*Overweight ≥85th Percentile Weight for Length) @ Timepoint: (%, (N/Total))*	
Birth	7.4 (14/189)
6 months	26.1 (47/180)
12 months	27.4 (48/174)
2 years	40.5 (72/178)
*Obese (≥95th Percentile Weight for Length) @ Timepoint: (%, (N/Total))*	
Birth	2.1 (4/189)
6 months	11.1 (20/180)
12 months	12.6 (22/175)
2 years	20.2 (36/178)
*Weight for Length Z scores @ Timepoint: (Mean ±SD)*	
Birth	−.51±1.10
6 months	.40±1.10
12 months	.31±1.24
2 years	.83±1.31
*<5th Percentile Weight for Age @ Timepoint: (%, (N/Total))*	
Birth	5.2 (10/194)
6 months	1.7 (3/181)
12 months	5.5 (10/182)
2 years	2.2 (4/181)
*Waist circumference >90th percentile (%, (N/Total))*	
2 years	8.3 (13/156)
*Maternal Postnatal Body Mass Index (Mean± SD)*	
12 months-2 years	28.3±6.3
*Maternal Postnatal Body Mass Index, Category*	
*<25*	32.5 (62/191)
*25–30*	34.0 (65/191)
*≥30*	33.5 (64/191)
*Breast-feeding and Age of Solids Introduction*	
Any Breast-feeding (BF) @ 4-6 wks postpartum (%, (N/Total))	91.2 (175/192)
Exclusive BF @ 4–6 weeks (%, (N/Total))	37.4 (71/190)
Any BF @ 6 months (%, (N/Total)	70.0 (126/180)
Any BF @ 12 months (%, (N/Total)	39.4 (67/170)
Age of Solids Introduction (months) (Mean*±* SD)	5.2±1.5

### Infant Feeding Characteristics

A high percentage (91.2%) of the mothers were breast-feeding (BF) at 4–6 weeks, 70.0% at 6 months, and 38.8% at 12 months of age (Table 1). A much lower 37.4% was exclusively BF at 4–6 weeks [18]. The mean age of introduction of any solid foods in this population was 5.2±1.5 months (Table 1) although as we have previously reported the introduction of teas and water occurred much earlier [18].

### Exposure to Maternal Depression and Child Weight Parameters

**Figure 1:**
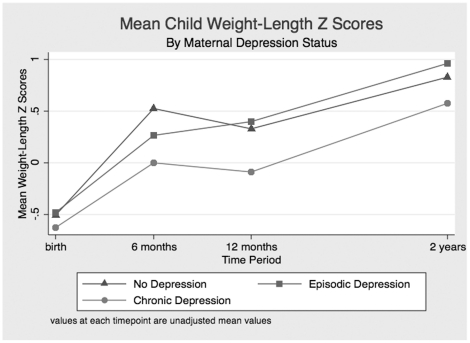
Mean Weight-Length Z Score by Maternal Depression Status. Values at each timepoint are unadjusted mean values.

**Figure 2:**
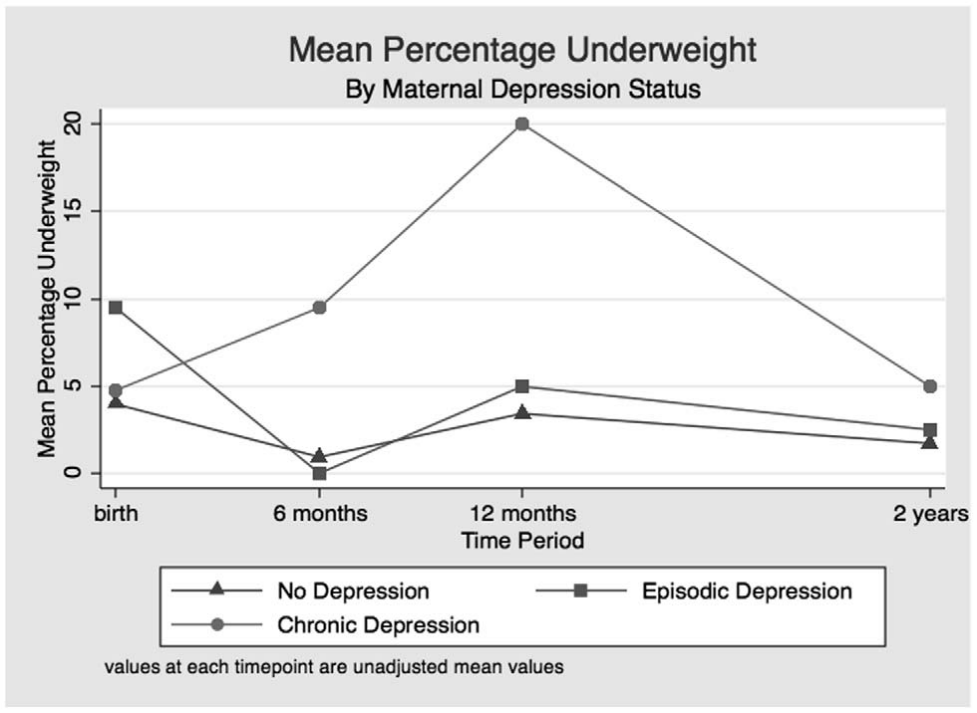
Percentage Underweight by Maternal Depression Status. Values at each timepoint are unadjusted mean values.

Exposure to chronic maternal depression was associated with decreased frequency of overweight at 6, 12 and 24 months, although the results did not achieve statistical significance (Table 2). Similarly, children exposed to chronic depression had decreased frequency of obesity at 6, 12 and 24 months, although this also did not reach statistical significance. Infants exposed to chronic maternal depression had lower weight/length Z-scores at 6, 12 and 24 months compared with those who were unexposed or those exposed episodically. Similarly, failure to thrive was more prevalent at all timepoints among infants and toddlers exposed to chronic maternal depressive symptoms in comparison with those unexposed (Table 2). No associations were found between mothers who had clinical depression (as determined from MINI) and child growth, or between maternal depressive symptoms and infant waist circumference >90^th^ percentile at age 2 years (results not shown). Figures 1 and 2 describe the relationships between exposures to chronic depression, episodic depression, and growth (as determined by weight for length z score) and risk for failure to thrive. Mean values at each time point are plotted for each group.

**Table 2:** Weight Parameters in Relation to Maternal Depression Symptom Status at Study Timepoints.

	Chronic Depression	Episodic Depression	Not Depressed	p-value
	% (N/Total),	% (N/Total),	% (N/Total),	
Variable	Mean ±SD	Mean ±SD	Mean ±SD	
*Overweight (≥85th Percentile Weight for Length)* *(%, (N/Total))* *@ Timepoint:*				
Birth	4.8 (1/21)	9.5 (4/42)	6.6 (8/121)	0.74
6 months	19.1 (4/21)	22.0 (9/41)	29.3 (34/116)	0.47
12 months	10.0 (2/20)	23.7 (9/38)	32.2 (37/114)	0.096
2 years	25.0 (5/20)	40.0 (16/40)	43.5 (50/115)	0.30
*Obese (≥95th Percentile Weight for Length)* *(%, (N/Total))* *@ Timepoint:*				
Birth	0.0 (0/21)	7.1 (3/42)	0.8 (1/121)	0.04
6 months	4.8 (1/21)	9.8 (4/41)	13.0 (15/116)	0.52
12 months	0.0 (0/20)	10.5 (4/38)	15.7 (18/115)	0.14
2 years	15.0 (3/20)	22.5 (9/40)	20.9 (24/115)	0.79
*Weight for Length Z scores (Mean ±SD)* *@ Timepoint*				
Birth	−0.62±0.93	−0.48±1.37	−0.51±1.01	0.88
6 months	0.016±1.10	0.22±1.09	0.53±1.05	0.07
12 months	−0.089±0.99	0.40±1.09	0.34±1.33	0.31
2 years	0.58±1.31	0.96±1.50	0.83±1.24	0.57
*Underweight (<5th Percentile Weight for Age)* *(%, (N/Total))* *@ Timepoint*				
Birth	4.8 (1/21)	9.5 (4/42)	4.0 (5/126)	0.38
6 months	9.5 (2/21)	0.0 (0/41)	0.9 (1/117)	0.01
12 months	20.0 (4/20)	5.0 (2/40)	3.3 (4/120)	0.01
2 years	5.00 (1/20)	2.5 (1/40)	1.7 (2/118)	0.65

### Independent Risk Factors for Infant Weight Gain

Logistic mixed modeling revealed that exposure to chronic depression within the first two years of life was associated with a decreased weight-for-length z-score (Coef −0.43, 95%CI −0.89—0.02) compared with no exposure or exposure only episodically (Table 3). In models adjusted for maternal postnatal BMI, maternal age, Mexican versus Central American ethnicity, any breast-feeding at 6 months of age, gestational age and birth weight-for-length z-score, decreased weight-for-length z score was similarly associated with chronic depression (Coeff = −0.48, 95%CI −0.94—0.01) (Table 4) In multivariate models, decreased maternal age was also associated with a lower weight-for-length z-score (Coef  = −0.03; 95%CI −0.06—0.002) and increased gestational age was associated with a higher weight-for-length z score (Coeff = 0.011, 95%CI 0.005-0.211). Increased maternal BMI neared significance for higher infant weight-for-length z scores (Coeff = 0.02, −0.008-0.05).

**Table 3:** Mixed Model of the Association Between Maternal Depression and Weight for Length Z Scores in Children from Birth to 2 Years of Age (n = 186 mother-child pairs).

Variable	Coeff (95%CI)	P value
No Depressive Symptoms	0.00	
Episodic Depression	−0.05 (−0.40−0.29)	0.76
Chronic Depression	−0.42 (−0.88−0.03)	0.07
Study Visit Time (6mo, 12 mo or 2 years)	0.03 (0.02−0.04)	<0.01

**Table 4:** Multivariate Mixed Model* of the Association Between Maternal Depression and Weight-for-Length Z Scores in Children from Birth to 2 years (n = 166 mother-child pairs).

Variable	Coeff (95%CI)	P value
No depressive symptoms	0.00	
Episodic depression	−0.09 (−0.45−0.27)	0.64
Chronic depression	−0.48 (−0.94—0.01)	0.045
Any breastfeeding @ 6mo	−0.25 (−0.60−09)	0.15
Mexican ethnicity	−0.09 (−0.41−0.22)	0.55
Maternal postnatal BMI	0.02 (−0.0008−0.05)	0.06
Maternal age	−0.03 (−0.06—0.002)	0.04
Gestational age	0.11 (0.005−0.21)	0.04
Birth Weight-for-Length Z score	0.10 (−0.03−0.24)	0.14
Study Visit Time, months	0.03 (0.02−0.04)	<0.01

Similarly, exposure to chronic maternal depression was associated with increased risk for failure to thrive/underweight (OR 11.81, 95%CI 1.70–82.61) (Table 5). In multivariate models, maternal chronic depression increased risk for failure to thrive (OR 12.12, 95%CI 1.86–78.78) and any breastfeeding at 6 months was associated to increased risk although results neared statistical significance (OR 8.77, 95%CI 0.82–93.74) (Table 6). Exposure to chronic depression was also associated with decreased risk for overweight in bivariate (OR 0.29, 95%CI 0.09–0.98) and multivariate models (OR 0.28, 95%CI 0.03–0.92) in comparison with children not exposed to maternal depressive symptoms or those exposed episodically (Table 7–8). Increased maternal postnatal BMI and infant gestational age were also associated with increased risk for overweight (OR 1.07, 95%CI 1.01–1.13 for maternal postnatal BMI; OR 1.33, 95%CI 1.04–1.70 for infant gestational age).

**Table 5:** Logistic Mixed Model of the Association between Maternal Depression and Risk for Failure to Thrive (Weight for Age Percentile <5th) from 6 months to 2 years (n = 186 mother-child pairs).

Variable	Odds Ratio 95% Confidence Interval	P Value
No Depressive Symptoms		1.00
Episodic Depression	1.17 (0.17−8.25)	0.87
Chronic Depression	11.81 (1.70−82.16)	0.01
Study Visit Time, months	1.01 (0.93−1.08)	0.91

**Table 6:** Multivariate Mixed Model* of the Association between Maternal Logistic Depression and Risk for Failure to Thrive (Weight for Age <5th percentile) 6 months to 2 years (n = 170 mother-child pairs).

Variable	Odds Ratio 95% Confidence Interval	P Value
No depressive symptoms	1.00	1.00
Episodic depression	0.31 (0.02−4.17)	0.38
Chronic depression	12.12 (1.86−78.78)	<0.01
Maternal postnatal BMI	1.02 (0.90−1.16)	0.37
Mexican ethnicity	0.84 (0.17−4.08)	0.83
Any breastfeeding @ 6mo	8.77 (0.82−93.74)	0.07
Maternal age	0.99 (0.84−1.15)	0.87
Gestational age	0.81 (0.43−1.56)	0.54
Birth weight Z score	0.52 (0.15−1.73)	0.83
Study visit time, months	1.00 (0.92−1.08)	0.96

*Models are adjusted for all variables listed in the table.

**Table 7:** Logistic Mixed Model of the Association between Maternal Depression and Child Overweight (Weight-for-Length Percentile ≥85th) from 6 months to 2 years (n = 186 mother-child pairs).

Variable	Odds Ratio 95% Confidence Interval	P Value
No Depressive Symptoms	1.00	
Episodic Depression	0.68 (0.29−1.60)	0.38
Chronic Depression	0.29 (0.09−0.98)	0.046
Study Time Visit (6mo, 12 mo or 2 years)	1.06 (1.02−1.09)	<0.01

**Table 8:** Multivariate Logistic Mixed Model* of the Association between Maternal Depression and Risk for Child Overweight (Weight-for-Length Percentile ≥85th) from 6 months to 2 years (n = 166 mother-child pairs).

Variable	Odds Ratio 95% Confidence Interval	P Value
No depressive symptoms	1.00	
Episodic depression	0.61 (0.26−1.44)	0.26
Chronic depression	0.28 (0.03−0.92)	0.04
Maternal postnatal BMI	1.07 (1.01−1.13)	0.02
Mexican ethnicity	0.75 (0.36−1.57)	0.43
Any Breastfeeding @ 6mo	0.81 (0.36−1.82)	0.60
Maternal age	0.95 (0.89−1.01)	0.12
Gestational age	1.33 (1.04−1.70)	0.03
Birth weight Z score	1.33 (0.96−1.84)	0.08
Study Time Visit, months	1.05(1.02−1.09)	<0.01

*Models are adjusted for all variables listed in the table.

When exposure to pre- and post-natal depression was evaluated separately in relation to weight-for-length z-scores and risk for failure to thrive, we did not find differences between the models that evaluated exposures to prenatal and postnatal depression separately and those that evaluated episodic versus chronic exposure to maternal depression. In both models, increased risks for lower weight-for-length z-scores and failure to thrive were associated with exposure to chronic depression compared with infants who were unexposed, exposed to episodic depression, or exposed to prenatal or postnatal depression alone. We also did not find any effect using an interaction term modeling time in relation to maternal depression status so we removed the interaction term from the multivariate models.

## Discussion

Ours is the first study to longitudinally evaluate the relationship between weight gain patterns in the first two years of life in Latino children and exposures to maternal depression in the perinatal period. In contrast to other studies that have evaluated these relationships [8], [10], our study had measures of depressive symptoms from the prenatal and postnatal period, facilitating our ability to evaluate the impact of chronic depression that spans the perinatal period.

Other studies have evaluated relationships between depression and growth either cross-sectionally [8] or measured depression at only one timepoint (pre- or post-natally), possibly explaining the lack of association [10]. Another factor that could explain the discrepancy in findings compared with our study is the time period of evaluation of postpartum depression. Ertel et al. found increased overall adiposity at age 3 was related to postpartum depression [9]. However postpartum depression was measured late in the postpartum (at 6 months), in contrast to our measurements at 4-6 weeks which likely would have a different impact on the growing infant and could differentially impact feeding practices. We also did not find any association between measures of abdominal obesity (waist circumference) and maternal depressive symptoms in contrast to Ertel et al., although they used a different measurement of central adiposity - skinfolds (subscapular [SS] and triceps [TR]) to derive a SS:TR ratio [9].

Our results also revealed that infants exposed to prenatal depression in utero or to chronic depression were more likely to have failure to thrive in the first 2 years of life compared with children who were unexposed. Previous studies have found that high prenatal cortisol is associated with prematurity and lower birthweight through hyperactivation of the HPA axis [25]. Neonates of depressed mothers have greater right frontal EEG activation, lower vagal tone, behavioral issues, and higher cortisol and norepinephrine levels, suggesting that the infants of depressed mothers have neurobehavioral dysregulation as early as birth that could impact weight gain patterns in the first years of life [26]. We did not evaluate the onset of depressive symptoms in our cohort, so we do not know if the presence of depressive symptoms at certain timepoints in pregnancy differently impact fetal growth rate and infant growth patterns. A longitudinal study from Nigeria found that infants of mothers with depressive symptoms were more likely to have failure to thrive from the 6^th^ week of life until 9 months of age compared with unexposed infants [27]. However, this study did not assess prenatal depressive symptoms and only assessed postnatal depressive symptoms at 6 weeks of age.

While we did not find any differences in exposure to depression and breastfeeding duration, previous investigators have reported that women with depressive symptoms in the postpartum period have decreased breastfeeding duration and increased breast-feeding difficulties [28]. Mothers with depressive symptoms are reported to be at greater risk for nonresponsive feeding styles [29]. One limitation of our study was that the frequency of clinical depression in our cohort was relatively low (4.0% prenatally and 4.3% at 4–6 weeks). We found no association between weight gain in the infants and exposure to maternal clinical depression in comparison with those exposed only to prenatal or postnatal depression. It is possible, however that the mothers with chronic depression had more severe depressive symptoms (although not severe enough to meet the criteria for clinical depression as per the M.I.N.I); we found that these mothers tended to score higher on both the CESD and EPDS compared with mothers who had episodic depression, although the results were not statistically significant. Similarly, we did not investigate the role of migration stress or immigration status as a possible contributor to depressive symptoms because of the sensitive nature of questioning in this area. However, our population of women was relatively homogenous in terms of language use (Spanish – 92.6%) and birth status (foreign born – 93.5%) so theses factors may not have differentially contributed to increased stress in a sub-set of our population.

We did not investigate the relationship between medication use for treatment of depression on infant outcomes because of the low percentage of medication use in our population, although this could be a fruitful area for future research. Similarly, we also did not examine or the role of stress hormones such as cortisol or growth hormones on patterns of weight gain.

From a clinical standpoint, infants of women with chronic depression should be evaluated for failure to thrive and the mothers should potentially be provided with additional nutritional support in addition to any needed psychological or psychiatric intervention. Interventions should be proactive given the behavioral and developmental risk factors associated with failure to thrive. Our results indicate that it is the combined exposures in utero and maternal-child interactions in the postpartum period will also contribute to failure to thrive, in contrast to other studies which have only assessed the role of prenatal depression or postnatal on child weight gain [3], [8].
